# Comparison of Pre-Analytical FFPE Sample Preparation Methods and Their Impact on Massively Parallel Sequencing in Routine Diagnostics

**DOI:** 10.1371/journal.pone.0104566

**Published:** 2014-08-08

**Authors:** Carina Heydt, Jana Fassunke, Helen Künstlinger, Michaela Angelika Ihle, Katharina König, Lukas Carl Heukamp, Hans-Ulrich Schildhaus, Margarete Odenthal, Reinhard Büttner, Sabine Merkelbach-Bruse

**Affiliations:** 1 Institute of Pathology, University Hospital Cologne, Cologne, Germany; 2 Institute of Pathology, University Hospital Göttingen, Göttingen, Germany; Medical University Vienna, Center for Brain Research, Austria

## Abstract

Over the last years, massively parallel sequencing has rapidly evolved and has now transitioned into molecular pathology routine laboratories. It is an attractive platform for analysing multiple genes at the same time with very little input material. Therefore, the need for high quality DNA obtained from automated DNA extraction systems has increased, especially to those laboratories which are dealing with formalin-fixed paraffin-embedded (FFPE) material and high sample throughput. This study evaluated five automated FFPE DNA extraction systems as well as five DNA quantification systems using the three most common techniques, UV spectrophotometry, fluorescent dye-based quantification and quantitative PCR, on 26 FFPE tissue samples. Additionally, the effects on downstream applications were analysed to find the most suitable pre-analytical methods for massively parallel sequencing in routine diagnostics. The results revealed that the Maxwell 16 from Promega (Mannheim, Germany) seems to be the superior system for DNA extraction from FFPE material. The extracts had a 1.3–24.6-fold higher DNA concentration in comparison to the other extraction systems, a higher quality and were most suitable for downstream applications. The comparison of the five quantification methods showed intermethod variations but all methods could be used to estimate the right amount for PCR amplification and for massively parallel sequencing. Interestingly, the best results in massively parallel sequencing were obtained with a DNA input of 15 ng determined by the NanoDrop 2000c spectrophotometer (Thermo Fisher Scientific, Waltham, MA, USA). No difference could be detected in mutation analysis based on the results of the quantification methods. These findings emphasise, that it is particularly important to choose the most reliable and constant DNA extraction system, especially when using small biopsies and low elution volumes, and that all common DNA quantification techniques can be used for downstream applications like massively parallel sequencing.

## Introduction

Formalin-fixation and paraffin-embedding (FFPE) is still the method of choice for preserving clinical tumour specimens. As a consequence, most molecular pathology routine laboratories perform mutational analysis for the diagnosis of different types of cancer and the evaluation of therapy options on FFPE tissue samples [1]. New methods such as massively parallel (or next generation) sequencing are now transitioning into molecular pathology laboratories [2]–[4]. The advantage of massively parallel sequencing is the capability of analysing multiple genes at the same time with little input material. Therefore the need for high quality FFPE DNA extracts has increased over the last years [5].

Especially to those molecular pathology laboratories with a high sample throughput, automated DNA extraction systems are essential. A robust, efficient and sensitive automated DNA extraction system for FFPE tissue samples is needed to reduce hands-on time to a minimum, to allow for sample tracking and to guarantee a reproducible sample quality. Further, more and more FFPE samples are small biopsies, which are now analysed by massively parallel sequencing [6]. Thus, an automated DNA extraction system that gives the highest DNA quantity and quality as possible, without inhibiting the downstream applications, is needed. Many studies have evaluated and improved DNA extraction methods from FFPE samples [7]–[9], however most studies compared manual extraction methods with each other or only one automated extraction system with manual DNA extraction [10]. This is the first study comparing different automated DNA extraction systems with FFPE material.

Additionally, an accurate and reliable DNA quantification system is necessary for a good and constant massively parallel sequencing performance [11]. There are few studies comparing DNA quantification methods from FFPE samples, but the results are varying [11]–[14]. Some studies state that quantitative PCR (qPCR) is the most accurate quantification method and that spectrophotometric analysis is the least reliable method for the detection of intact double stranded DNA [15]. Other studies showed that a combination of fluorescent dye-based quantification systems such as the Qubit 2.0 fluorometer with a spectrophotometric system like the NanoDrop 2000c spectrophotometer is the most accurate method for assessing the quantity and purity of DNA and can also be used for the massively parallel sequencing workflow [11]. However, none of the studies gave a comprehensive comparison of more than three quantification methods using UV spectrophotometry, fluorescent dye-based quantification and qPCR.

This study aimed to compare and evaluate five automated DNA extraction systems as well as five DNA quantification methods and their impact on downstream applications to find the most suitable pre-analytical workflow for massively parallel sequencing in routine diagnostics.

## Materials and Methods

### Samples

26 samples, varying in size from small biopsies to large resections, were selected from the registry of the Institute of Pathology of the University Hospital Cologne, Germany. All samples were routinely formalin-fixed and paraffin embedded (FFPE). The FFPE tissue samples were obtained as part of routine clinical care with verbal informed consent from each patient and under approved ethical protocols complied with the Ethics Committee of the Medical Faculty of the University of Cologne, Germany. All FFPE samples were made anonymous and the Ethics Committee waived the need for written informed consent.

### Automated DNA extraction systems

10 of the FFPE tissue samples collected in the year 2013 were used for the comparison of five automated DNA extraction systems, the BioRobot M48, the QIAcube and the QIAsymphony SP all from Qiagen (Hilden, Germany), the Maxwell 16 from Promega (Mannheim, Germany) and the InnuPure C16 from Analytik Jena (Jena, Germany).

For all five systems, 10 µm thick sections were cut from the FFPE tissue blocks and deparaffinised. The tumour areas were macrodissected from an unstained slide with a sterile scalpel. A previously marked haematoxylin and eosin-stained slide served as reference. For each method and tissue block the same tumour area and number of sections were used.

The tissues were further processed using the commercial available FFPE kits for each automated DNA extraction system. The five kits were MagAttract DNA Mini M48 Kit (BioRobot M48, Qiagen), QIAamp DNA FFPE Tissue Kit (QIAcube, Qiagen), QIAsymphony DNA Mini Kit (QIAsymphony SP, Qiagen), innuPREP FFPE DNA Kit – IPC16 (InnuPure C16, Analytik Jena) and the Maxwell 16 FFPE Plus LEV DNA Purification Kit (Maxwell 16, Promega). DNA isolation was performed according to manufacturer's instructions. All DNA extraction systems work with magnetic bead based purification systems, except for the QIAcube (Qiagen) which has a spin column based DNA purification. All tissues were lysed with proteinase K overnight and the elution volume was set to 50 µl. DNA was eluted in Tris-HCl (pH 7, 6) on all systems except the QIAcube (Qiagen) where the supplied ATE buffer was used.

### Quantification of DNA purity and concentration

The genomic DNA extracted with each system was quantified in duplicates with the NanoDrop 2000c spectrophotometer (Thermo Fisher Scientific, Waltham, MA, USA). 1 µl of the extracted DNA was used for measuring the DNA concentration and the absorbance ratio at 260/280 nm for evaluation of the purity of each sample.

Additionally, each DNA sample was quantified in duplicates with the Qubit 2.0 fluorometer (Life Technologies, Darmstadt, Germany). The Quant-iT dsDNA HS Assay (Life Technologies) was used according to the manufacturer's instructions.

To further assess the DNA quality, 5 µl of each DNA extract were examined on an ethidium bromide stained 1% agarose gel.

### Assessment of PCR amplifiable fragment length

The amplifiable fragment length of the DNA extracts was assessed by PCR amplification of three different fragments of the *glyceraldehyde-3-phosphate dehydrogenase* (*GAPDH*) gene. DNA amplification was carried out in a 25 µl PCR reaction containing 20 ng DNA template measured with the NanoDrop 2000c spectrophotometer (Thermo Fisher Scientific), 200 pM forward and reverse primer (Table 1), 10× PCR buffer, 1.5 mM MgCl_2_, 200 µM dNTP and 1 U Platinum Taq polymerase (Invitrogen, Darmstadt, Germany). The cycling conditions on a T3000 thermocycler (Biometra, Goettingen, Germany) were as follows: 94°C for 3 minutes; 40 cycles at 94°C for 40 seconds, 62°C for 40 seconds, 72°C for 35 seconds; and hold for 5 minutes at 72°C. The quality of the PCR products was assessed by the QIAxcel capillary electrophoresis (Qiagen).

**Table 1:** Sequences and product sizes of PCR and qPCR primers.

Name	Gene	Primer sequence (5'–3')	Product size (bp)
GAPDH-201-F	*GAPDH*	GCTCCCACCTTTCTCATCCA	201
GAPDH-201-R	*GAPDH*	GTCTTCTGGGTGGCAGTGAT	201
GAPDH-404-F	*GAPDH*	CATGGTATGAGAGCTGGGGA	404
GAPDH-404-R	*GAPDH*	GTCCACCACTGACACGTTG	404
GAPDH-614-F	*GAPDH*	GAGTCCACTGGCGTCTTCA	614
GAPDH-614-R	*GAPDH*	GTCTGCAAAAGGAGTGAGGC	614
HFE-F	*HFE*	GCCATAATTACCTCCTCAGGCAC	234
HFE-R	*HFE*	ATGGATGCCAAGGAGTTCGAACC	234
EGFR-21-F	*EGFR*	CGGATGCAGAGCTTCTTCCC	275
EGFR-22-R	*EGFR*	AGGCAGCCTGGTCCCTGGTG	275

F: Forward.

R: Reverse.

### Assessment of DNA quality by multiplex PCR amplification

The extracted and quantified FFPE DNA was subsequently amplified in a multiplex PCR with 102 amplicons of 14 different genes using an Ion AmpliSeq custom DNA panel from Life Technologies. The amplicon sizes were 125–175 bps and were especially designed for FFPE material with the Ion AmpliSeq Designer (Life Technologies). 21 ng of extracted FFPE DNA, determined by the Qubit 2.0 fluorometer (Life Technologies), was used for multiplex PCR amplification, according to the manufacturer's instructions. The quality of the PCR product was assessed by the QIAxcel capillary electrophoresis (Qiagen).

### DNA quantification methods

With the 16 remaining FFPE samples, which were collected and extracted in the years 2005 2013, five quantification methods were tested and compared. DNA was quantified by UV absorbance, fluorescent dye-based quantification and qPCR.

The DNA of 12 of the FFPE samples was isolated as described above using the BioRobot M48 (Qiagen), following the manufacturer's instructions. The DNA of the other 4 FFPE samples was from 2010 and older and was isolated manually with the QIAamp DNA Mini Kit (Qiagen), according to the manufacturer's protocol. To assess the DNA quality, 5 µl of each DNA extract were examined on an ethidium bromide stained 1% agarose gel.

The 16 DNA extracts were quantified in duplicates with the NanoDrop 2000c spectrophotometer (Thermo Fisher Scientific), the Quant-iT dsDNA HS Assay on the Qubit 2.0 fluorometer (Life Technologies), the QuantiFluor dsDNA Sample Kit on the QuantiFluor-ST fluorometer (Promega) and the Quant-iT PicoGreen dsDNA reagent (Life Technologies) on the LightCycler 480 Instrument (Roche, Mannheim, Germany). All protocols were performed according to the manufacturer's instructions. Additionally, a qPCR for amplification of the *hemochromatosis* (*HFE*) gene was performed on a CFX96 real-time system (Bio-Rad). The reaction volume was 20 µl, consisting of 1 µl DNA template, 20 pM forward and reverse primer (Table 1) and 10 µl SsoFast EvaGreen Supermix (Bio-Rad). The cycling conditions were as follows: 94°C for 5 minutes; 55 cycles at 94°C for 30 seconds, 60°C for 30 seconds, 72°C for 30 seconds. A standard curve using cell line derived DNA in concentrations ranging from 0,195 ng/µl to 50 ng/µl was also included in the qPCR to determine the sample concentrations.

### Assessment of PCR amplification

The accuracy of the DNA quantification methods and the impact on downstream applications were first examined by PCR amplification of the *epidermal growth factor receptor* (*EGFR*) gene. The 25 µl PCR reactions contained 20 ng DNA template, calculated for each quantification method and sample, 200 pM forward and reverse primer (Table 1), 10× PCR buffer, 2 mM MgCl_2_, 200 µM dNTP and 1 U Platinum Taq polymerase (Invitrogen). The cycling conditions on a T3000 thermocycler (Biometra) were as follows: 94°C for 3 minutes; 41 cycles at 94°C for 40 seconds, 60°C for 40 seconds, 72°C for 35 seconds; and hold for 5 minutes at 72°C. The quality of the PCR products was assessed by the QIAxcel capillary electrophoresis (Qiagen).

### Impact of DNA quantification methods on the results of amplicon-based massively parallel sequencing

To further assess the impact of DNA quantification methods on parallel sequencing, two additional FFPE samples from 2013 were extracted with the Maxwell 16 (Promega) and quantified in duplicates with the NanoDrop 2000c spectrophotometer (Thermo Fisher Scientific), the Qubit 2.0 fluorometer (Life Technologies) and by qPCR representative for each quantification technology. The quantified FFPE DNA was subsequently amplified in the same custom designed multiplex PCR with 102 amplicons from Life Technologies as described above. 5 ng, 15 ng, 25 ng and 35 ng of DNA calculated for each quantification method and sample were used for the multiplex PCR amplification resulting in 24 samples. The quality of the PCR product was assessed by the QIAxcel capillary electrophoresis (Qiagen). After multiplex PCR, libraries for each sample were generated by adapter ligation and target enrichment with the Ion AmpliSeq Library Kit 2.0 (Life Technologies) as described previously [16]. The size of the obtained library was assessed by the QIAxcel capillary electrophoresis (Qiagen). Each sample library was measured with the Qubit 2.0 fluorometer, diluted to a concentration of 15 nM and sequenced on a MiSeq benchtop sequencer (Illumina, San Diego, USA) according to the manufacturer's instructions. Data analysis was performed using an in-house software and the data were visualised with the Integrative Genome Viewer (IGV) (Broad Institute, Cambridge, USA). The mean and the standard deviation (SD) were calculated for the number of variants and the allele frequency of mutations above a 5% background threshold.

## Results and Discussion

### Automated DNA extraction systems

#### Quantification of DNA purity and concentration

**Figure 1:**
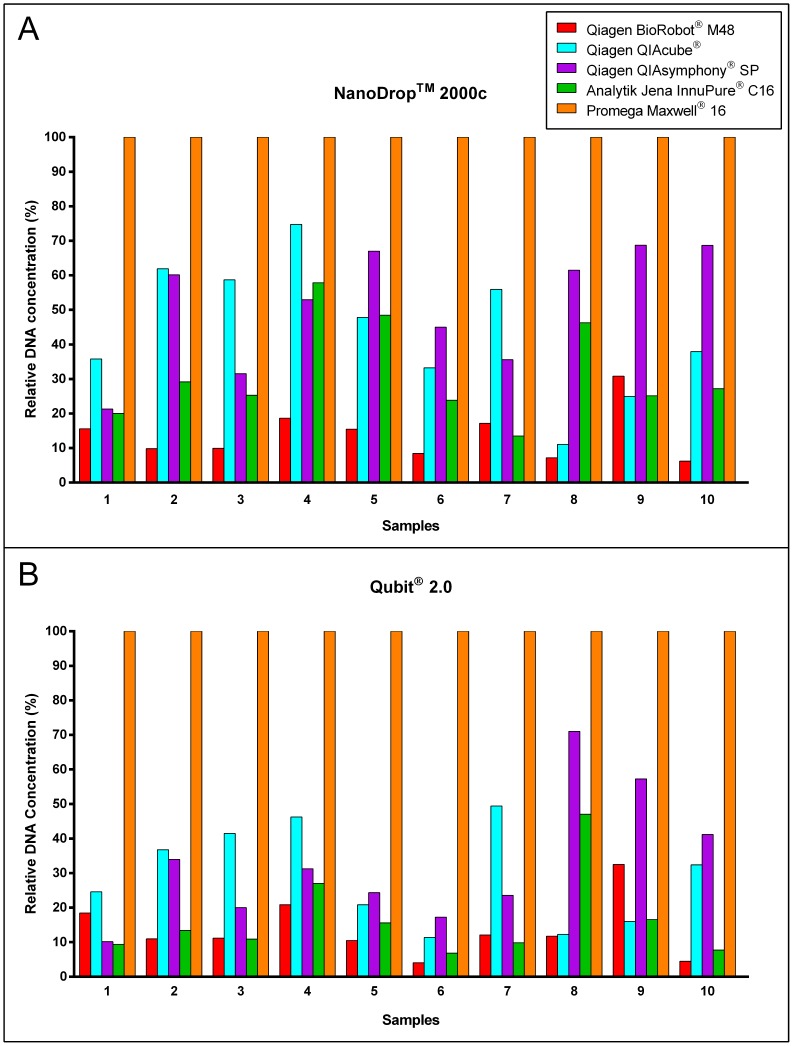
Comparison of five automated DNA extraction systems. Illustration of relative DNA concentrations of samples 1–10 measured by the NanoDrop 2000c spectrophotometer (A) and Qubit 2.0 fluorometer (B). The concentrations of the Maxwell 16 extracts were set to 100% for each sample.

For the comparison of the five automated DNA extraction systems 10 FFPE tissue samples were extracted with each system (Table S1). The relative DNA concentrations (percentage of the highest concentration obtained by the Maxwell 16 extracts) for each method and sample is shown in figure 1. The extracted DNA was quantified by the NanoDrop 2000c spectrophotometer (Figure 1 A) and the Qubit 2.0 fluorometer (Figure 1 B). Additionally, the purity of each sample was determined by measuring the absorbance ratio at wavelength 260/280 nm (Table S1). As illustrated in figure 1, the amount of DNA extracted by the Maxwell 16 system was the highest in all samples, independent of tumour size. The DNA quantity obtained from the BioRobot M48 was the lowest in 9 samples when measured with the NanoDrop 2000c spectrophotometer and in 8 samples when measured with the Qubit 2.0 fluorometer. A 1.3–24.6-fold difference between the concentration of the Maxwell 16 extracts and the extracts of the other systems could be seen. The second highest DNA concentrations were obtained by the QIAcube and the QIAsymphony SP followed by the InnuPure C16 and the BioRobot M48. The DNA concentrations determined by the Qubit 2.0 fluorometer showed in all samples an even higher difference in DNA quantity between the extracts of the Maxwell 16 and the extracts of the other instruments whereas the order of the instruments from highest concentration to lowest stayed more or less the same.

Samples 5 and 6, which were small biopsies, showed the highest difference between their Qubit 2.0 fluorometer and NanoDrop 2000c spectrophotometer values of the BioRobot M48, the QIAcube, the QIAsymphony SP and the InnuPure C16 extracts in relation to the Maxwell 16 extracts. One explanation could be that these samples have a very low DNA concentration of below 10 ng/µl and it is commonly known that values below 10 ng/µl are less accurate with the NanoDrop 2000c spectrophotometer [17].

The purity ratio 260/280 nm of all samples except sample 5 and 6 were for all five automated DNA extraction systems around 1.8–2.0. Samples 5 and 6 showed values over 4 in the BioRobot M48 extracts and values of 2.55 and 2.37 in the InnuPure C16 extracts.

#### Assessment of DNA quality by downstream applications

**Figure 2:**
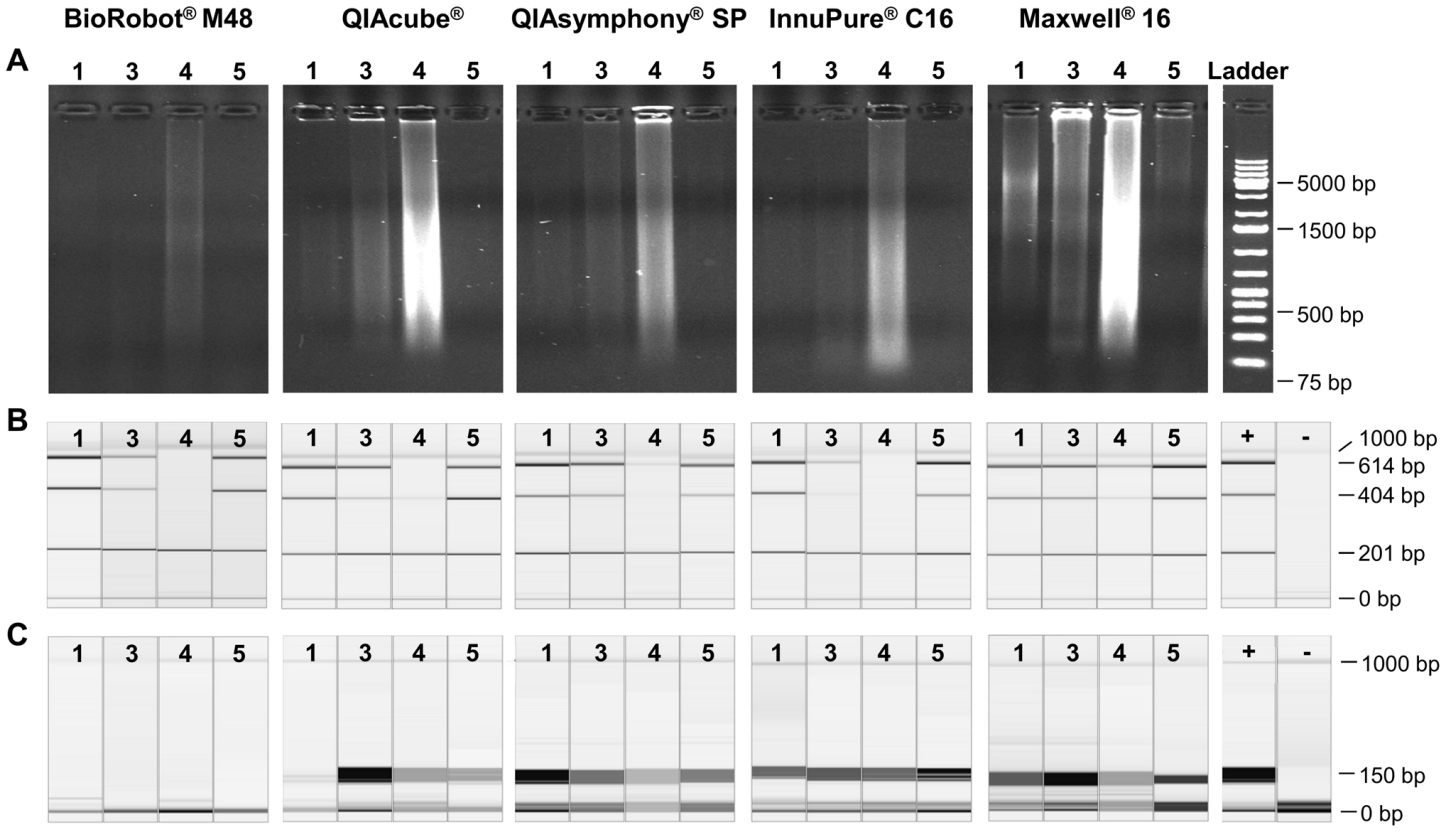
Assessment of DNA quality obtained from each extraction method and impact on downstream applications. (A) Electrophoretic pattern of DNA extracts 1, 3, 4 and 5 of each extraction method on a 1% agarose gel. Ladder indicates a 1 kb DNA ladder as molecular weight size marker (B) 201 bp, 404 bp and 614 bp amplified DNA fragments of the *GAPDH* gene for sample 1, 3, 4, 5, of each extraction method. + indicates a positive control and – a negative control. (C) 125–175 bp multiplex PCR product for sample 1, 3, 4 and 5 of each extraction method. + indicates a positive control and – a negative control.

First, the DNA extracts were loaded on a 1% agarose gel to visualise the quality and quantity of the sample and to determine the amount of degradation (Figure 2 A). The DNA extracts from the Maxwell 16 showed the highest DNA quantity as already seen in figure 1. Additionally, less degradation and DNA of higher molecular weight could be observed in comparison to the extracts from the other instruments.

To determine the usability of the five DNA extraction systems for downstream applications, PCRs yielding in 201 bp, 401 bp and 614 bp fragments of the *GAPDH* gene to assess the maximal amplifiable fragment length were performed on samples 1, 3, 4, and 5 representative for small, medium and large tissue samples. Additionally, a 102 amplicon custom designed multiplex PCR from Ion AmpliSeq was used.The results for samples 1, 3, 4 and 5 are shown in figure 2. The 201 bp *GAPDH* fragment could be amplified in all extracts. The 404 bp and 614 bp *GAPDH* fragments could be amplified successfully in samples 1 and 5 of all extraction methods (Figure 2 B). Sample 3, showing some degradation, was well amplified in the QIAcube, QIAsymphony SP and Maxwell 16 extracts and only marginally amplified in the BioRobot M48 and InnuPure C16 extracts. Sample 4 showing high degradation was only well amplified in the Maxwell 16 extracts, due to the fact that the Maxwell 16 extract had more and higher molecular weight DNA present.

The assessment of the DNA quality by the custom designed Ion AmpliSeq PCR showed that the BioRobot M48 DNA extracts inhibit the Ion AmpliSeq PCR (Figure 2 C). This can be due to the high salt concentration present in the samples [18]. A 1∶2 dilution of the 50 µl BioRobot M48 eluates with Tris-HCl solved this problem and the Ion AmpliSeq PCR was no longer inhibited. However, dilution of samples is not feasible for DNA extracts from small biopsies as the DNA concentration should be kept as high as possible for downstream applications to minimise sequencing artefacts [19]. For all other DNA extracts the Ion AmpliSeq PCR worked well, except for sample 1 of the QIAcube extracts.

In summary, the Maxwell 16 seems to be the superior system for the extraction of DNA from FFPE material. Our results demonstrate that the Maxwell 16 extracts have a 1.3–24.6-fold higher DNA concentration than the other systems and a DNA quality which is most suitable for downstream applications. The second best results were obtained with the QIAcube and QIAsymphony SP. The quality of the InnuPure C16 and BioRobot M48 extracts is less suitable for downstream applications used in this study.

In table 2 the catalogue prices for each instrument and the material costs per sample are listed. The cheapest instrument is the InnuPure C16 with around 15,000 € followed by the QIAcube and the Maxwell 16 with around 20,000 €. The most expensive instrument is the QIAsymphony SP with around 85,000 € and the BioRobot M48 with around 55,000 €, whereas the BioRobot M48 is no longer commercially available. The material costs per sample are the cheapest with the QIAcube Kits (2.89–3.20 €), followed by the BioRobot M48 Kit (3.60 €) and dependent on the kit size the QIAsymphony SP Kits (3.77–6.93 €). The material costs per sample with the Maxwell 16 Kit (6.02 €) and the InnuPure Kits (5.63–6.17 €) are the most expensive. Thus, the DNA extraction with the QIAcube, one of the second best instruments, is the cheapest.

**Table 2:** Comparision of instrument prices and material costs per sample for five automated DNA extraction systems.

Extraction system	Instrument price (€)	Costs per sample (€)
QIAsymphony SP (Qiagen)	from ∼85,000	3.77–6.93
QIAcube (Qiagen)	from ∼20,000	2.89–3.20
Maxwell 16 (Promega)	from ∼20,000	6.02
InnuPure C16 (Analytik Jena)	∼15,000	5.63–6.17
BioRobot M48 (Qiagen)	∼55,000*	3.60

All costs are based on the recent catalogue pricings.

*: The instrument is no longer commercially available.

∼: Approximately.

Thus far, no other studies are published comparing different automated FFPE DNA extraction systems. Only one study compared the Maxwell 16 extraction system to manual DNA extraction. This study showed that the amount of DNA obtained from the Maxwell 16 is significantly lower in comparison to the AllPrep FFPE DNA/RNA Mini Kit (Qiagen) but there was no significant difference in downstream PCRs. Furthermore, the Maxwell 16 extracts were the most consistent for PCR products smaller than 402 bp [10]. In contrast to our study, the Maxwell 16 FFPE Tissue LEV DNA Purification Kit was used which might have lower DNA output compared to the PLUS version.

When comparing the results of maximum amplifiable fragment length from our study to other studies, which used manual DNA extraction, it can be seen that the Maxwell 16 extracts perform equally good [20] or in some cases even better [8], [12].

### DNA quantification methods

**Figure 3:**
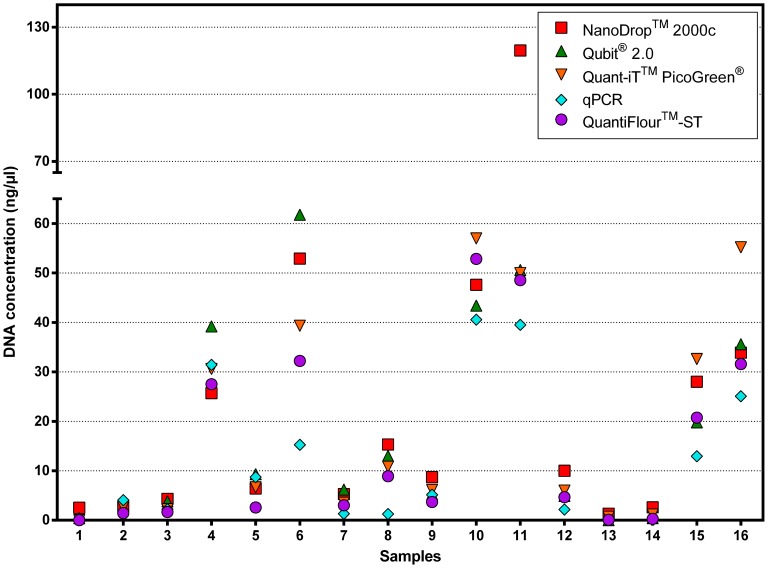
Results of the five DNA quantification measurements. Distribution of DNA concentrations of samples 1–16 measured by each of the five quantification method.

The DNA of 16 previously extracted FFPE tissue blocks were analysed with five different quantification methods. Some of these extracts were prepared manually using the QIAamp DNA Mini Kit. According to previous studies DNA extracts from the QIAamp DNA Mini Kit are of good DNA quality and quantity [8]. The results of the five DNA quantification methods are shown in figure 3 and table S2.

**Figure 4:**
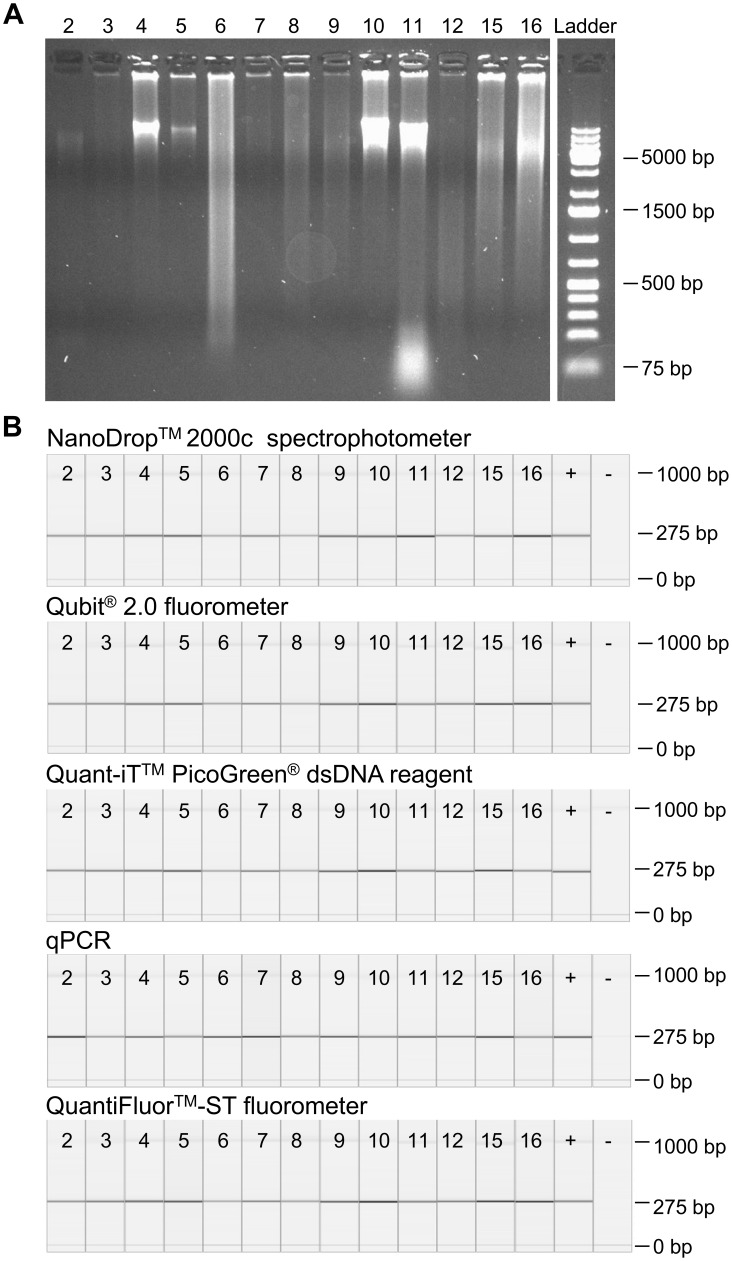
Impact of DNA quantification methods on downstream applications. (A) Electrophoretic pattern of 13 DNA extracts on a 1% agarose gel. (B) Amplified 275 bp fragment of the *EGFR* gene. 20 ng of sample DNA determined by each quantification method was used for PCR amplification. + indicates a positive control, - a negative control.

Concerning the level of DNA concentration there was no uniformity in comparing the five methods. In samples 4, 5, 6 and 7 the Qubit 2.0 fluorometer measured the highest DNA concentrations, in sample 10, 15 and 16 the Quant-iT PicoGreen dsDNA reagent measurement gave the highest values, in sample 2 the qPCR and in all other samples the NanoDrop 2000c spectrophotometer estimated the highest DNA concentrations. This is in contrast to other studies where the NanoDrop 2000c spectrophotometer always overestimated the DNA concentration [15]. In general, DNA concentrations determined by the different quantification methods were more diverse at a concentration higher than 10 ng/µl, especially in samples 6 and 11. In samples 6 and 11 we saw differences of almost 50 ng/µl and 70 ng/µl between the lowest and the highest measured concentrations. Relating these findings to a 1% agarose gel of the DNA extracts (Figure 4 A) it could be seen that sample 6 and 11 have a high level of degraded DNA. In degraded DNA samples the DNA is fragmented into smaller DNA fragments. Sedlackova et al. [15] showed that fragmentation does not affect the concentration measured with the NanoDrop 2000c spectrophotometer and that this method might even overestimate the DNA concentration due to the fact that the NanoDrop 2000c spectrophotometer also measures single-stranded DNA and oligonucleotides present. Further they state that fluorescent-dye based quantification methods and qPCR are significantly affected by DNA fragmentation: The higher the fragmentation the lower the DNA concentration. This effect can also be seen in our study.

The DNA concentration estimated by qPCR for samples 8, 15 and 16 was lower than with the other methods although the DNA was not much degraded and was not affected by DNA fragmentation (Figure 3, 4 A). Previous studies have shown that the accuracy of DNA quantification by qPCR can also be affected by other reasons. Standard curves used in qPCRs are in general error prone and might lead to wrong results [21]. Further, qPCRs should be designed for multiple fragment lengths and reference genes to minimise the quantification errors [22]–[24]. Another problem might be the differences in amplification efficiency between the DNA samples and standards due to different tissues used and potential sample contaminations. [21], [24], [25]. All these facts might lead to under- or overestimation of the DNA concentration. In our study the qPCR results seem to underestimate the DNA concentration.

#### Assessment of PCR amplification

To assess the quality of the DNA quantification methods PCR amplification of a 275 bp fragment of the *EGFR* gene was analysed. 20 ng of DNA from 13 of the 16 tissue blocks, calculated by the five quantification methods were used for PCR amplification (Figure 4 B). In each sample a 275 bp fragment could be amplified, no difference in PCR amplification could be seen. Only the band intensity differed between the samples but there was not any tendency towards a better performance of one of the quantification methods. These findings demonstrate that in a well-established and robust PCR setting, the amount of input material is less responsible for the result of downstream application. Even samples 6 and 11 with a discrepancy of 50 and 70 ng/µl could be amplified with all estimated amounts of DNA.

#### Impact of DNA quantification methods on the results of amplicon-based massively parallel sequencing

**Figure 5:**
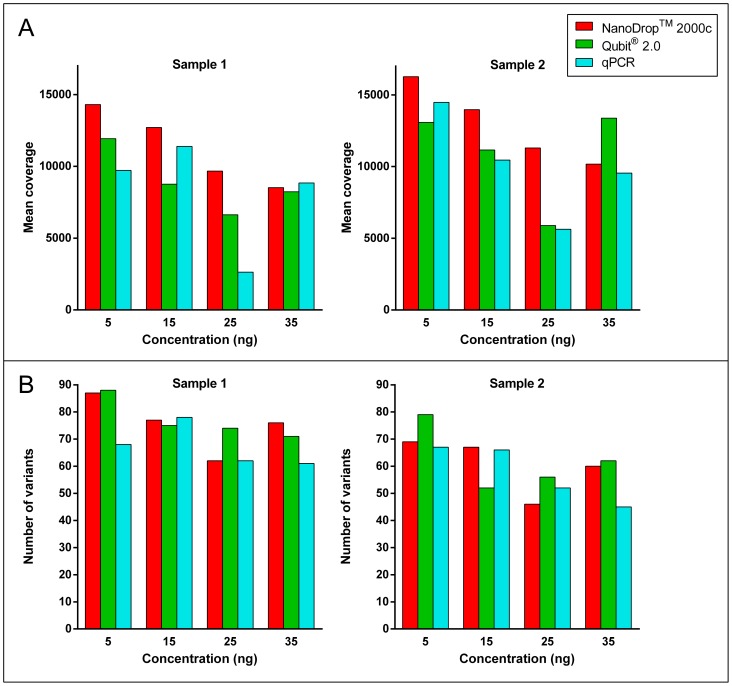
Impact of DNA quantification methods on massively parallel sequencing. (A) Mean amplicon coverage determined by in-house software for each sample, quantification method and amount of DNA used for multiplex PCR amplification. (B) Number of all variants called by in-house software for each sample, quantification method and starting material used.

The usefulness of each DNA quantification method for downstream applications was further assessed by massively parallel sequencing to see if different quantification methods have an impact on mutation calling, the number of variants and the mean coverage. Therefore two additional samples were extracted with the Maxwell 16 and quantified. The results for sample 1 were 155 ng/µl (NanoDrop 2000c spectrophotometer), 84.2 ng/µl (Qubit 2.0 fluorometer) and 20.9 ng/µl (qPCR). The results for sample 2 were 86.7 ng/µl (NanoDrop 2000c spectrophotometer), 46.8 ng/µl (Qubit 2.0 fluorometer) and 10.7 ng/µl (qPCR). PCR products could be seen for each sample, quantification method and concentration. The libraries were sequenced on a MiSeq benchtop sequencer with a cluster density of 1154 K/mm^2^, a Q30 score of 96.5% and a cluster passing filter value of 94.8%. These results are in the upper range for massively parallel sequencing on a MiSeq benchtop sequencer according to the Illumina specifications [26]. The data analyses revealed that independently from the quantification method and the amount of DNA used the massively parallel sequencing worked well when using an extensively validated routine workflow (Figure 5). Until now 2000 samples were routinely analysed in our laboratory with the Ion AmpliSeq custom DNA panel used in this study. This workflow was also used in the study of Ihle et al., where 72 samples were analysed with the same Ion AmpliSeq custom DNA panel [16].

The best results for the mean coverage were obtained with the concentrations determined by the NanoDrop 2000c spectrophotometer (Figure 5 A) and the highest values were achieved with a DNA input of 5 ng for both samples. The lowest results were obtained with a DNA input of 25 ng measured with the Qubit 2.0 fluorometer and qPCR. The mean coverage of all samples was over 2000× and therefore suitable for analysis in a routine laboratory setting.

The numbers of all variants determined by the in-house software were also the highest in the 5 ng samples (Figure 5 B). However, when setting a background threshold of 5% allele frequency, 92±1.0% (Mean ± SD) of all variants of sample 1 were below 5% allele frequency and 78±4.7% of these variants were transitions C>T or A>G. In sample 2 85±3.3% of all variants were below 5% allele frequency and 70±6.4% of these variants were transitions. In FFPE samples transitions are often due to fixation artefacts [27], [28]. Fixation artefacts are a common problem when using FFPE samples especially when analysing small biopsies with low tumour content [19], [29]. Do et al. showed that in massively parallel sequencing 70–90% of all mutations under 10% are fixation artefacts [30]. Other studies showed that a threshold of 4–5% allele frequency [4] as well as a sequencing coverage per amplicon of at least 80× is necessary to reduce false positive variants [31]. These findings are consistent with the results of our study. In our study a threshold of 5% allele frequency was needed for reducing false positive variants and it could be seen that the background noise was increased with a DNA input of only 5 ng.

Concerning the impact on mutation calling above a 5% threshold, it could be seen that independently of the quantification method or amount of DNA input, mutations could be detected in all samples at almost the same allele frequency. In sample 1 the *TP53* mutation p.H179L could be detected with a frequency of 27±1.6% (mean±SD) and the *ALK* mutation p.L1204P with a frequency of 6±0.6%. In sample 2 the *KRAS* variant p.Q61H was present at 11±0.9%, the *TP53* variant p.R110L at 6±0.7% and the *ALK* mutation p.L1204P at 5±0.5%. The *KRAS* and *TP53* mutations could be verified by Sanger sequencing whereas the validation did not work for the *ALK* mutations as they were around the detection limit of this technology. Nevertheless, all mutations were detected in 12 individual multiplex PCR reactions for each sample with similar allele frequencies and were determined reliable. Taking all data into account a DNA input of 15 ng yielded the best results in variant detection and mean coverage.

This study showed that all five quantification methods can be used for downstream applications. Even the NanoDrop 2000c spectrophotometer measurements can be used for subsequent sample analysis with massively parallel sequencing. A previous study by Hadd et al. [4] also used the NanoDrop 2000c spectrophotometer for DNA quantification for targeted next generation sequencing. Nevertheless prior to massively parallel sequencing fluorescent dye-based quantification methods are commonly used as they are considered to be the easiest, most reliable and cost effective methods [3]. From our point of view qPCR is the least feasible method as it is time consuming, expensive and not practicable in routine laboratories with a high sample throughput, which is also stated by other studies [13], [15]. Additionally, our study showed that samples with a low amount of amplifiable DNA, determined by qPCR, could still be amplified by a well-established PCR and the DNA sample could still be used for mutation detection. In general, low amount of DNA present is a limiting factor for downstream applications and is more influential than an inaccurate quantification method, especially for massively parallel sequencing and can result in the detection of fixation artefacts [13], [31]. Therefore it is very important to use a DNA extraction method with a high DNA yield like the Maxwell 16, especially when dealing with small biopsies.

## Conclusions

This study showed that while implementing massively parallel sequencing in the routine laboratory it is not only important to focus on the sequencing platform and the data analysis pipeline but also on the pre-analytical steps. It is essential to choose the most reliable and constant FFPE DNA extraction system for sample preparation, especially when using small biopsies and low elution volumes in routine diagnostics. In this study the Maxwell 16 from Promega turned out to be the system with the highest DNA quantity and quality among the five automated DNA extraction systems compared and gave the best results in downstream applications. The QIAcube and QIAsymphony SP both from Qiagen were the second best automated DNA extraction systems followed by the InnuPure C16 from Analytik Jena and the BioRobot M48 from Qiagen.

The comparison of the five DNA quantification systems showed intermethod variations but all methods could be used to estimate the right amount for PCR applications and for massively parallel sequencing. The NanoDrop 2000c spectrophotometer showed the best results for massively parallel sequencing in the mean coverage, but there was only a slight difference in mutation detection. The qPCR seemed to underestimate the DNA concentration. Altogether, our study has shown that in a molecular pathology routine laboratory it is important to decide upon one quantification method, to validate the chosen method and always to use the same method.

## Supporting Information

Table S1 **Comparison of five automated DNA extraction system.**
(XLS)

Table S2 **Comparison of five DNA quantification methods.**
(XLS)
